# Epidemiology of Ticks and Tick-Borne Pathogens in Domestic Ruminants across Southern African Development Community (SADC) Region from 1980 until 2021: A Systematic Review and Meta-Analysis

**DOI:** 10.3390/pathogens11080929

**Published:** 2022-08-18

**Authors:** Mpho Tawana, ThankGod E. Onyiche, Tsepo Ramatla, Sibusiso Mtshali, Oriel Thekisoe

**Affiliations:** 1 Unit for Environmental Sciences and Management, North-West University, Potchefstroom 2531, South Africa; 2 Department of Veterinary Parasitology and Entomology, University of Maiduguri, Maiduguri 600230, Nigeria; 3 Foundational Research and Services, South African National Biodiversity Institute, National Zoological Gardens, Pretoria 0001, South Africa; 4 University of Limpopo, Private Bag X1106, Sovenga 0727, South Africa

**Keywords:** SADC, ticks and tick-borne pathogens, *Amblyomma*, *Boophilus*, *Haemaphysalis*, *Hyalomma*, *Rhipicephalus*, *Anaplasma*, *Ricketssia*, *Babesia*, *Theileria*

## Abstract

Ticks are hematophagous ectoparasites that are capable of infesting a wide range of mammals, including domestic animals, ruminants, wildlife, and humans across the world, and they transmit disease-causing pathogens. Numerous individual epidemiological studies have been conducted on the distribution and prevalence of ticks and tick-borne diseases (TBDs) in the Southern African Developing Community (SADC) region, but no effort has been undertaken to synchronize findings, which would be helpful in the implementation of consolidated tick control measures. With the aim of generating consolidated pooled prevalence estimates of ticks and TBDs in the SADC region, we performed a systematic review and meta-analysis of published articles using the PRISMA 2020 guidelines. A deep search was performed on five electronic databases, namely, PubMed, ScienceDirect, Google Scholar, AJOL, and Springer Link. Of the 347 articles identified, only 61 of the articles were eligible for inclusion. In total, 18,355 tick specimens were collected, belonging to the genera *Amblyomma*, *Haemaphysalis*, *Hyalomma*, and *Rhipicephalus* (including *Boophilus*) across several countries, including South Africa (n = 8), Tanzania (n = 3), Zambia (n = 2), Zimbabwe (n = 2), Madagascar (n = 2), Angola (n = 2), Mozambique (n = 1), and Comoros (n = 1). The overall pooled prevalence estimate (PPE) of TBPs in livestock was 52.2%, with the highest PPE in cattle [51.2%], followed by sheep [45.4%], and goats [29.9%]. For bacteria-like and rickettsial TBPs, *Anaplasma marginale* had the highest PPE of 45.9%, followed by *A. centrale* [14.7%], *A. phagocytophilum* [2.52%], and *A. bovis* [0.88%], whilst *Ehrlichia ruminantium* had a PPE of 4.2%. For piroplasmids, *Babesia bigemina* and *B. bovis* had PPEs of 20.8% and 20.3%, respectively. *Theileria velifera* had the highest PPE of 43.0%, followed by *T. mutans* [29.1%], *T. parva* [25.0%], and other *Theileria* spp. [14.06%]. Findings from this study suggest the need for a consolidated scientific approach in the investigation of ticks, TBPs, and TBDs in the whole SADC region, as most of the TBDs are transboundary and require a regional control strategy.

## 1. Introduction

Ticks are a major group of the hematophagous arthropods that are a veterinary and public health concern, resulting in major financial suffering for the agricultural sector [1]. Primarily, ticks are normally pathogen transmitters and are found in bushes, forests, and semi-arid areas [2]. The availability and behaviour of ticks highly depend on the climatic conditions of a geographical area [3,4,5]. Subsequently, the alteration of the climatic conditions of an area can lead to a high prevalence of ticks and tick-borne disease, as observed in Tunisia [6].

The Southern African Developing Community (SADC) region has tick species that belong to three families, including *Argasidae*, *Ixodidae*, and *Nuttalliellidae* [7,8,9,10,11]. *Ixodidae*, which are known as hard ticks, are blood-sucking arthropods of amphibians, avians, reptiles, and mammals and they include various genera, such as *Ixodes*, *Amblyomma*, *Dermacentor*, *Haemaphysalis*, *Hyalomma*, and *Rhipicephalus* [12]. *Argasidae* is a family of soft ticks that does not lack scutum, as compared to the hard tick family; it has four genera, including *Argas*, *Carios*, *Ornithodoros*, and *Otobius* [10]. Lastly, the family *Nuttalliellidae* has only one tick species known as *Nuttalliella namaqua*, which shares similar characteristics to *Argasidae* and *Ixodidae* ticks [13,14].

Generally, adult ticks target medium- and large-sized hosts (animals and sometimes humans) for blood meal, whilst larvae and nymphs target small hosts for their survival [15]. These ticks are known to be global vectors of microorganisms infecting both animals and humans, such as bacteria, helminths, protozoal, ricketssial, and viral pathogens [16,17].

Ticks are distributors of tick-borne diseases (TBDs) amongst tropical and subtropical regions of the world [18,19,20]. Based on epidemiology, tick-borne diseases vary according to unequally shared space and time in various nations that depend specifically on the availability of certain tick species [21]. In urban and suburban areas, small rodents, hedgehogs, and shrews are well-known reservoirs of *Ixodes* ticks species, which transmit tick-borne pathogens [TBPs] [22]. This suggests that ticks are available in suburban green areas and the diversity and rate of tick-borne diseases in those areas might be equivalent to rural areas when quantified [23]. The TBPs may be more accurately detected by the use of more sensitive molecular techniques, such as conventional polymerase chain reaction (PCR), nested PCR (nPCR), real-time polymerase chain reaction (qPCR), reverse transcription-polymerase chain reaction (RT-PCR), and reverse line blot hybridization (RLB) [24,25]. Numerous TBPs, including *Anaplasma* spp., *Babesia* spp., *Ehrlichia* spp., *Ricketssia* spp., and *Theileria* spp., have been documented as significant pathogens of domestic ruminants, including cattle, goats, and sheep, and are further transmitted by ticks within individual SADC region countries [26,27,28,29].

For effective control measures against ticks and TBPs to be successful, it is pertinent that the distribution of ticks and TBPs within that particular geographical area is known. Therefore, this study was undertaken to generate consolidated data of tick abundance, as well as TBPs’ prevalence and distribution, from blood and feeding ticks collected from domestic ruminants in the SADC region, using systematic review and meta-analysis.

## 2. Results

### 2.1. Literature Search and Eligible Studies

**Figure 1:**
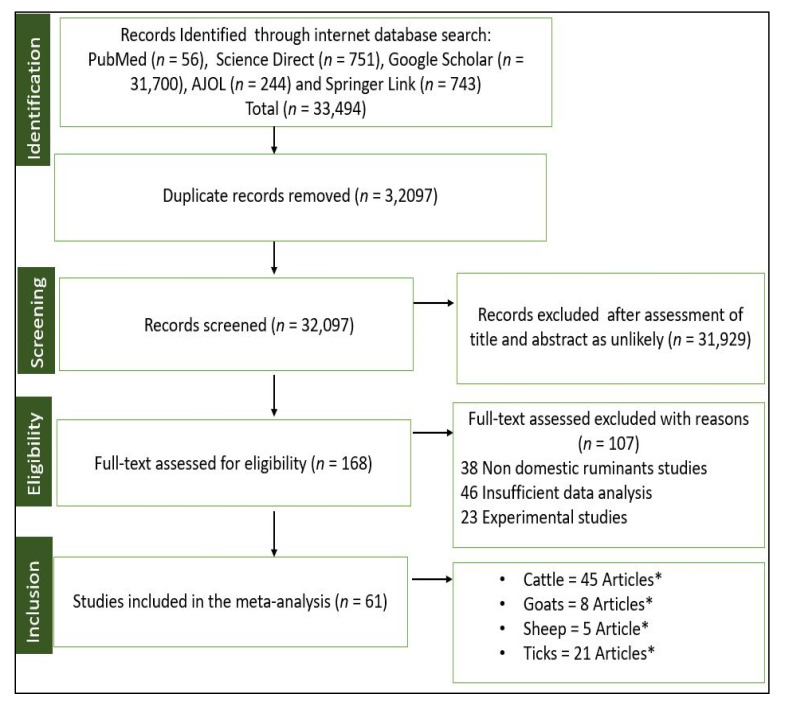
Flow chart of included studies, according to Preferred Reporting Items for Systematic Reviews and Meta-Analyses guidelines. * Whilst n = 61 articles were used for the meta-analysis, in some cases, the same article has published data from different ruminants hosts as well as ticks; hence, the total number of articles may appear as if it is more than 61.

A total search of 33,494 articles was initially identified through PubMed (n = 56), Science Direct (n = 751), Google Scholar (n = 31,700), AJOL (n = 244), and Springer Link (n = 743). After the removal of duplicates, 32,097 articles remained. Of these, 31,929 articles were excluded after assessing the titles and abstracts. The remaining 168 full articles were then assessed for eligibility, and 107 from the following groups were excluded: (i) studies on non-domestic ruminants (n = 38); (ii) studies with insufficient data analysis (n = 46); and (iii) experimental studies (n = 23). Finally, 61 articles that reported on the prevalence of ticks and tick-borne pathogens detected from blood and tick samples of ruminants across the SADC region fulfilled the criteria for inclusion (Figure 1).

### 2.2. Characteristics of Eligible Studies

The occurrence of tick-borne pathogens was detected in three livestock ruminant groups, namely, cattle (n = 45), goats (n = 8), and sheep (n = 5), with a total of 12,693 examined blood samples, published in any of the SADC countries (Table 1). These studies were conducted in South Africa (n = 18); Tanzania (n = 10); Zambia (n = 7); Mozambique (n = 5); Angola (n = 4); Botswana (n = 2); and Zimbabwe (n = 1). The blood sample size ranged between 8 and 960 per study and the individual prevalence of tick-borne pathogens in different animal species ranged from 0.33% to 100% (Table 1). Additionally, 21 studies focused on the prevalence of TBPs in tick species, which were conducted in South Africa (n = 8), followed by Tanzania (n = 3), Zambia (n = 2), Zimbabwe (n = 2), Madagascar (n = 2), Angola (n = 2), Mozambique (n = 1), and Comoros (n = 1). The tick sample size ranged between 36 and 7364 per study and the individual prevalence of TBPs in different tick species ranged from 0.02% to 62.32% (Table 2).

### 2.3. Pooling, Heterogeneity, and Sub-Group Analysis

#### 2.3.1. Prevalence in Animals Based on Host, Study Years, Countries, Diagnostic Technique and Species of Tick-Borne Pathogens

The overall pooled prevalence estimate (PPE) of tick-borne pathogens (TBPs) in animals was 52.2% [(95% CI: 43.9–60.3%); Q = 2820.792; I^2^ = 98.33; *Q-p* = 0.609] (Table 2). The subgroup analysis by animal hosts revealed that the highest PPE in cattle was 51.2% [(95% CI: 42.9–59.4%); Q = 2491.04; I^2^ = 98.23; *Q-p* = 0.779], followed by sheep [45.4% (95% CI: 9.4–87.0%); Q = 146.22; I^2^ = 97.26; *Q-p* = 0.861], and goats had the lowest PPE [29.9% (95% CI: 7.3–69.9%); Q = 252.68; I2 = 97.23; *Q-p* = 0.325] (Table 2). Only one study was eligible for inclusion within the study period spanning from 1990 to 2000 with a PPE of 0.72%, while 21 studies were included within the period from 2011 to 2020 with a PPE of 57.3% (Table 2). Lastly, a PPE of 63.6% was observed in the period from 2001 to 2010. Seven studies used RLB diagnostic methods for the detection of tick-borne pathogens and these diagnostic methods recorded the highest pooled rate as 63.0% (95% CI: 42.0–80.0), followed by nPCR with 61.5 (95% CI: 45.6–75.2). PCR was recorded with a pooled prevalence of 43.6% (95% CI: 34.8–52.8) from twenty-eight studies. Statistics are recorded in Table 2. The forest plots that show the point estimates for the individual studies that describe the occurrence of *Anaplasma* *marginale*, *Babesia bigemina*, *B. bovis*, and *Theileria parva* are presented (Supplementary Figures S1–S4).

#### 2.3.2. Prevalence of Tick-Borne Pathogens in Different Species of Ticks 

Different TBPs were detected and reported in tick species collected from animals within the SADC region at varying prevalence (Table 3). The overall PPE of TBPs in tick populations was 10.7% [(95% CI: 5.7–19.1%); *Q* = 2132.53; I^2^ = 99.16; Q-*p* = 0.086] (Table 4). The subgroup analysis of bacterial TBPs is shown in Table 4.

#### 2.3.3. Prevalence of Tick-Borne Pathogens in Different Species of Ticks

Thirty tick species belonging to 8 genera, namely, *Rhipicephalus* (n = 10 species), *Amblyomma* (n = 9 species), *Heamaphysalis* (n = 3 species), *Boophilus* (n = 2 species), *Hyalomma* (n = 2 species), *Ixodes* (n = 2 species), *Margaropus* (n = 1 species), and *Otobius* (n = 1 species) were reported in the studies from different SADC countries (Table 5). The PPE of TBPs in 22 tick species from five different genera revealed pathogens to be more prevalent in the tick genus *Amblyomma* at 25.0% [(95% CI: 14.7–39.1%); *Q* = 598.25; I^2^ = 97.66; *Q-p* = 0.001], as compared to the genus *Rhipicephalus* at 11.7% [(95% CI: 4.7–26.2%); *Q* = 786.55; I^2^ = 98.47; *Q-p* = 0.000]. Other tick genera, including *Boophilus*, *Hyalomma*, and *Haemaphysalis*, expressed a PPE of 50%, 13.1%, and 10.5% for TBPs, respectively (Table 5).

**Figure 2:**
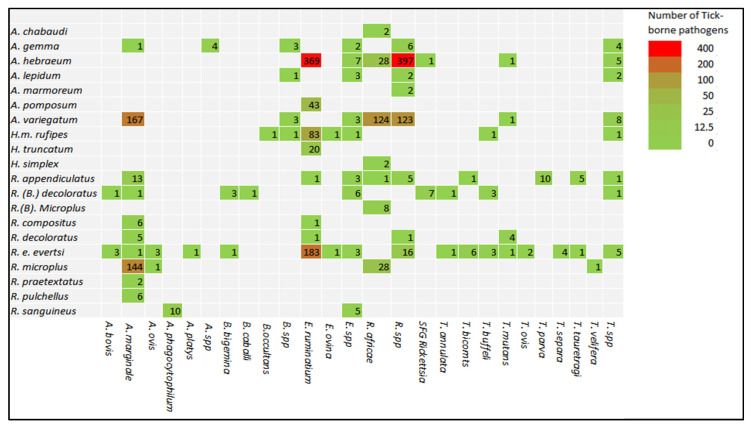
Heat map for linked relationship between ticks and tick-borne pathogens in the Southern African Developing Community region.

The analysis of tick species harbouring TBPs showed that *A. variegatum* was the species that harboured the most pathogen infections at 43.9% [(95% CI: 10.1–84.4%); *Q* = 250.42; I^2^ = 97.60; *Q-p* = 0.804]; followed by *R. microplus* at 15.4% [(95% CI: 1.1–75.5); *Q* = 173.07; I^2^ = 98.84; *Q-p* = 0.238]; then, *A. hebraeum* at 14.2% [(95% CI: 8.9–21.8%); Q = 64.23; I^2^ = 90.66; *Q-p* = 0.000]; *R. e. evertsi* at 7.4% [(95% CI: 1.1–35.8%); *Q* = 317.76; I^2^ = 98.74; *Q-p* = 0.011]; and *R. appendiculatus* at 5.5% [(95% CI: 2.8–10.4%); Q = 25.93; I^2^ = 73.00; *Q-p* = 0.000]. The percentages of tick species infected with TBPs, in descending order, was as follows: the *A. chabaudi* (100.0%), followed by *B. decoloratus* (60.0%), *B. microplus* (37.5%), *R. decoloratus* (28.6%), *R. pulchellus* (27.3%), *A. gemma* (25.3%), *A. lepidum* (19.1%), *A. marmoreum* (18.2%), *H. m. rufipes* (15.3%), *H. simplex* (10.5%), *R. praetextatus* (8.7%), *A. pomposum* (7.0%), *H. truncatum* (6.1%), *R. (B.) decoloratus* (5.9%), *R. sanguineus* (5.4%), *R. compositus* (3.9%), and *R.(B). microplus* (2.7%) [Table 6]. South Africa appeared to be the country with highest TBP prevalence in ticks, as compared to other countries in the SADC region, since most of their tick studies tested negative for tick-borne pathogens (Table 4, Figure 2).

#### 2.3.4. Assessment for Publication Bias in Studies Involving Domestic Ruminant Animals

**Figure 3:**
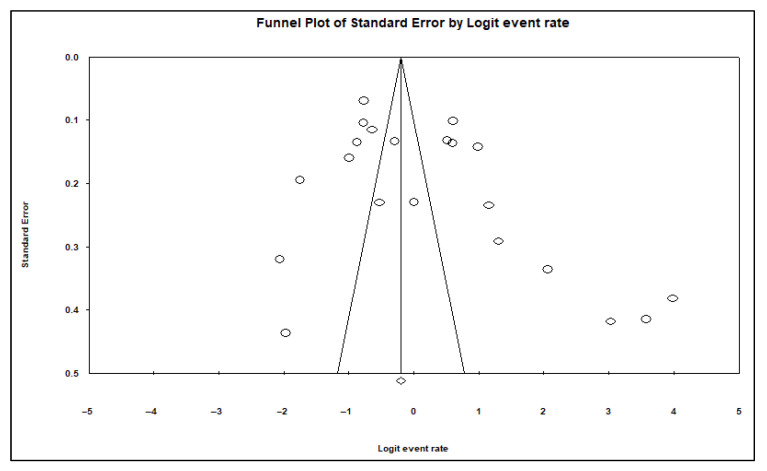
A funnel plot of subgroup studies tested reported positive detection of tick-borne pathogens in livestock for 2011–2021 year interval/period.

**Figure 4:**
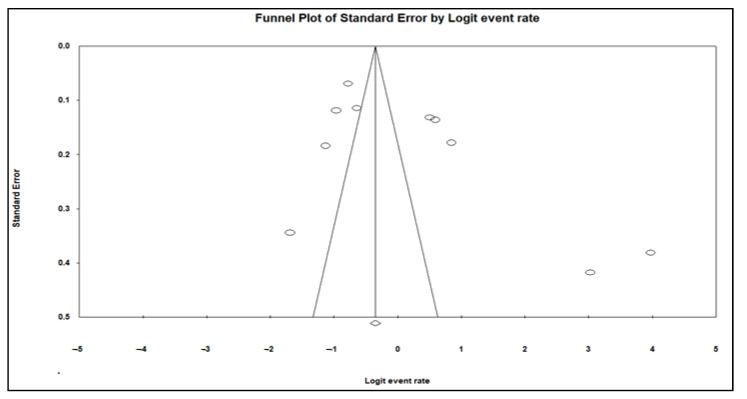
A funnel plot subgroup studies that positively detected tick-borne pathogens from livestock in Tanzania.

A funnel plot of standard error by logit event rate together with the Begg and Mazumdar rank correlation test were used to assess publication bias. Our data analyses showed no evidence of publication bias for almost all risk factors for animal studies, except for study period 2010–2021 (Table 2, Figure 3) and the country Tanzania, where significant bias was observed in regard to asymmetries of the funnel plots and *p*-values of 0.040 and 0.020, respectively (Table 2, Figure 4).

## 3. Discussion

### 3.1. Ticks

Tick identification and prevalence are significant factors in estimating the abundance of tick species in a population and in quantifying the prevalence of TBPs of public and animal health concern [91,92]. Tick-borne pathogens in African domestic ruminants are complex, with several tick species feeding on various animals to facilitate the transmission of numerous microorganisms [93].

In this study, a total of 26 tick species belonging to five genera of *Ixodidae*, namely, *Amblyomma*, *Hyalomma*, *Haemaphysalis*, and *Rhipicephalus* (including *Boophilus*), were recorded, with the *Rhipicephalus* genus being the most abundant. Similar findings were recorded in the Caribbean, where the genus *Rhipicephalus* was more prevalent as compared to other tick genera [94,95].

We also observed that *Amblyomma* ticks harboured more TBPs, as compared to the genus *Rhipicephalus*. These results are slightly similar to those reported in Kenya [96] and in Guinea and Liberia [97]. Specifically, *A. variegatum* was the most infected tick vector, while *R. appendiculatus* was the least; this finding is consistent with a previous report from Ethiopia [98]. In contrast, the authors of [99] reported a lower prevalence of tick-borne pathogens in *A. variegatum* in Oromia, Ethiopia. The high prevalence of tick-borne pathogens on *A. variegatum* ticks might suggest a public health concern to people living in the areas of sample collection if bitten by infected ticks, since this tick is known to transmit pathogens to both animals and humans [97].

There is a significant public and animal health risk associated with tick host preference, as they harbour zoonotic pathogens, including *Anaplasma* spp., *Babesia* spp., *Ehrlichia* spp., and *Rickettsia*, which can be transmitted at the interface between domestic animals, wildlife, and humans [100]. The overall PPE of TBPs in different ticks was below 20%, similar to previous reports in Turkey [101], Japan [102], and Greece [103]. However, higher prevalences of TBPs harboured by ticks was reported in Sudan [42.7%] [104] and Pakistan [35.1%] [105]. Spotted fever group is a causative agent of the rickettsioses belonging to the family *Rickettsiaceae* [106]. Ticks in the genus *Amblyomma* are known to be reservoirs and transmitters of *Rickettsia* spp. in cattle and humans in Africa [107]. According to Althaus et al. [108], *Rickettsia africae* is reportedly the most common species of rickettsial pathogens detected in ticks and humans in some parts of the African continent and is responsible for ~11% of African tick bite fever cases in tourists from South Africa.

### 3.2. Tick-Borne Pathogens in Different Animal Host

According to the analysed, published articles in the current study, TBPs have been detected from domestic animals in Angola, Botswana, Mozambique, South Africa, Tanzania, Zambia, and Zimbabwe with an overall PPE of 52.2%, which is a representative portion of the SADC region. This PPE is higher than that reported from Algeria (Central Africa), with prevalence of less than 20% [109], but was slightly lower than the 62.9% reported in Uganda (Eastern Africa) [110]. This high prevalence in SADC countries might be due to farm management, micro-climate patterns, tick distribution, and livestock breeds [111,112].

The PPE of TBPs in domestic ruminant as observed in this study was similar to that reported from the Caribbean, where cattle had a higher prevalence compared to sheep and goats [113]. In contrast, Bell-Sakyi et al. [114] reported TBPs from cattle to have a lesser prevalence compared to sheep and goats in Ghana. This variation in host prevalence might be associated with the livestock husbandry system practiced in most SADC countries, where cattle farming predominates that of small ruminants [115].

The genus *Anaplasma* with causal agents of anaplasmosis in cattle had a higher prevalence (45.6%) for the SADC countries in this study, as compared to the 5.3% and 22.6% that were previously reported from Uganda and Senegal, respectively [87,116]. Evidence of a similar prevalence to the current study was reported in Italy [50%] and Iran [77%] [117,118]. However, results according to species level revealed that *A. marginale* was the most prevalent species of *Anaplasma*, which is similar to reports from Turkey [119] and Iran [120]. In contrast, Mohammadian et al. [121] and Salehi-Guilandeh et al. [122] reported *A marginale* to be a less prevalent species in cattle, compared to other *Anaplasma* species in the west and northern regions of Iran.

*Babesia* are known to be pathogenic to cattle and can also pose a serious public health risk to humans [123]. The prevalence of *B. bigemina* was slightly higher as compared to *B. bovis*. Similar findings were observed in Brazil (34% for *B. bigemina* and 20.4% for *B. bovis*) [124] and Colombia (24.2% for *B. bigemina* and 14.4% for *B. bovis*) [125]. These findings can be explained by the observations of Kocan [126], who reported that higher temperatures hinder or terminate the synthesis of the *B. bigemina* pathogen in cattle ticks.

In the current study, the PPE of 4.2% for *E. ruminantium* is relatively similar to the 4.5% and 6.6% prevalences that were reported in Western Uganda [116] and Cameroon, respectively [127]. The PPE of the current study is, however, higher than the 0.5% and 1.1% prevalences reported in South-western Ethiopia and Nigeria, respectively [128,129]. The low prevalence might be due to colostrum and indigenous breed genes that can influence some level of resistance to *Ehrlichia* pathogens in domestic animals [130,131].

The species of the genus *Theileria* recorded from the studies used in this systematic review and meta-analysis includes *T. velifera* (43%) as the most prevalent, followed by *T. mutans* (29.1%), and *T. parva* (25.0%). In contrast, *T. velifera* was reported to be less prevalent than *T. parva* in Kenya and Uganda, with a prevalence of *T. velifera* at 1.3% and *T. parva* at 1.9% in Kenya, and *T. velifera* at 11.8% and *T. parva* 69.4% in Uganda. [132,133]. However, other studies reported *T. velifera* to be less prevalent than *T. mutans* in Southern Sudan [*T. velifera* 45.3%, *T. mutans* 73%] [134] and in Ethiopia [*T. velifera* 4%, *T. mutans* 8%] [135]. Our findings are in congruence with the results reported by Byaruhanga et al. [136], where the *T. velifera* [71.3%] prevalence was higher than *T. parva* [2.9%] in the Karamoja region, Uganda, and *T. velifera* [40%] was higher compared to *T. mutans* [25.7%] in Lambwe Valley, Kenya [137].

The findings on different molecular techniques revealed that the RLB technique was the most sensitive of all molecular-based methods. This is due to the fact that RLB assay is able to detect various TBPs from the same specimen simultaneously, unlike conventional PCR [138,139]. Similar results in accordance with current data were reported in a study detecting TBPs in Western Kenya using RLB and qPCR techniques, in which RLB was more sensitive compared to qPCR [93]. Furthermore, a study on small ruminant blood samples in Turkey reported that RT-PCR is more sensitive than the RLB method at detecting TBPs [24].

With regard to changes in prevalence over time, a declining trend in tick-borne pathogens prevalence was observed over the course of the 10-year intervals in our study, from the period 2001–2010 to the period 2011–2020 among cattle in SADC regions. The decline over time witnessed in this study suggests that there might have been some improvements by countries’ agricultural sectors (government and farmers) in tick, TBPs, and TBPDs control measures, such as proper use of acaricides during spraying and dipping.

### 3.3. Limitations

This regional approach provides statistical evidence and a clearer understanding of the spatial distribution of ticks and TBPs in the SADC region, which will assist in identifying countries in which there is a lack of scientific evidence. Future research may prioritize areas of emphasis for better understanding and consolidation of data to develop prevention and control strategies. We also highlighted the link between ticks and their pathogens by observing the interesting roles of this link from an epidemiological point of view. This systematic review and meta-analysis have multiple limitations, such as: (i) there was a small number of domestic ruminant studies conducted in the SADC; therefore, a subgroup analysis was impossible to conduct due to few eligible articles. This lack of small domestic ruminants data may be due to the influences of study location, farming activities of countries, belief or myths, sample sizes, availability of animals, and study designs of the individual studies. (ii) Most studies did not consider demographic characteristics, such as life stage of ticks, age of animals, or sex of animals and ticks. (iii) There was a large gap of study outputs between the study period from one publication from 1990–2000 and 21 publications in 2010–2020. (iv) There is also lack of published studies for TBPs using molecular diagnostic techniques from many countries, such as the Democratic Republic of Congo, Lesotho, Mauritius, Namibia, Seychelles, and Swaziland, and as a result, the analysis is not entirely representative of the entire SADC region. In Comoros and Madagascar, there were no articles published in which blood samples were screened for TBPs, while Botswana and Malawi did not have tick sample studies.

## 4. Materials and Methods

### 4.1. Search Strategy and Criteria

Literature searches were conducted on PubMed, Science Direct, and Google Scholar for articles published in the English language from 1 January 1980 until 22 March 2021, with content containing information on the prevalence or epidemiology of tick-borne pathogens across SADC countries in domestic animals. The search keywords were Ticks and Tick-borne pathogens in Southern Africa; Prevalence of “*Anaplasma*” “*Babesia*” “*Ehrlichia*” and/or “*Theileria*”. Keywords were used individually or in combination with the “AND” and/or “OR” operators (Table 6). None of the authors of the original studies were contacted for additional information and no attempt was made to retrieve unpublished articles. Titles and abstracts were scanned and relevant full text articles were downloaded and obtained through library resources and online platforms.

### 4.2. Inclusion and Exclusion Criteria

Articles were included only if they fulfilled the following inclusion criteria: cross sectional (prevalence) study conducted within the SADC region; involved invertebrate (ticks) and vertebrate host (cattle, goats and sheep); involved ticks and/or blood sample collection; exact total numbers and positive cases were clearly provided; involved the use of a molecular-based technique; sample size (>25 for enabling statistical calculations); written in English. Studies without the above-mentioned characters, such as reviews, experimental studies, non-domestic ruminant studies, insufficient data analyses, studies with lower sample sizes, and studies not written in English were all excluded.

### 4.3. Data Extraction

The data extraction protocol was prepared and evaluated by all authors. The data extraction protocol consists of the names of the authors and countries, hosts, total sample sizes, number of positive cases, estimated prevalence, species of blood pathogens, tick species, and molecular diagnostic technique. Moreover, studies that were conducted in more than one country and those that had both animal and tick studies simultaneously were separated accordingly.

Titles and abstracts derived through primary electronic search were thoroughly assessed for possibility of inclusion, based on the study type (prevalence of ticks and tick-borne pathogens of domestic ruminants) in the SADC region. From each eligible study, the following data were extracted, based on the performed software (Excel, Microsoft, 2016) format: author, study area, host, method used, study year, sample size, positive samples, different tick-borne pathogen species, and different tick species. All data were extracted using a standardized extraction form. For duplicate studies, only one study was selected. The extracted data were cross checked with the included papers, then modifications and editions of mistyped data were made when necessary.

### 4.4. Meta-Analytic Procedures

The current meta-analysis was conducted using Comprehensive Meta-Analysis software (CMA) version 3.0 software [140]. The pooled prevalence estimates and 95% CIs were calculated using random-effects models. Statistical heterogeneity between studies was measured by I^2^ statistic; I^2^ > 50% was defined as high heterogeneity [141]. Publication bias was measured using funnel plots to test the symmetry and the Begg’s and Mazumdar rank correlation test [142].

## 5. Conclusions

The highest PPE of TBPs in domestic animals in the SADC is recorded in Mozambique, which has warm subtropical and tropical climates, while the country of Botswana had the lowest PPE and a semi-arid climate. The major TBPs with high PPE in the SADC region for bacteria and piroplasmids include *A. marginale* and *T. velifera*. The RBL is more sensitive in detecting TBPs from blood samples as compared with other molecular techniques. The most prevalent TBP detected from ticks was *R. africae*, whilst *Rhipicephalus* ticks was the most prevalent in livestock. A higher prevalence rate of TBPs in the SADC was observed in domestic animal blood compared to tick species. Some of the TBDs are transboundary and require cooperation between neighbouring countries for their effective control. There is, therefore, a requirement for consolidated research into regional ticks and TBDs between SADC countries that would enable a united effort in documenting the prevalence and understanding of ticks and TBDs in the region.

## Figures and Tables

**Table 1:** Characteristics of all eligible studies reporting the occurrence of tick-borne pathogens in domestic ruminants across the Southern African Developing Community region.

Countries	Hosts	Sample Size	Total No. of Pathogens Detected	Pathogens Detected (No. of Positives, Prevalence (%)	Reference
Angola	Cattle	98	11	*A. platys* (n = 3; 3.06%), *A. capra* (n = 6; 6.12%), *A. phagocytophilum* (n = 2; 2.04%)	[30]
Angola	Cattle	76	51	*A. marginale* (n = 29; 38.15%), *Anaplasma* spp. (n = 6; 7.89%), *B. bigemina* (n = 2; 2.63%), *T. velifera* (n = 22; 28.95%), *Theileria* spp. (n = 6; 7.89%)	[31]
Angola	Goats	13	13	*A. ovis* (n = 13; 100.00%)	[30]
Angola	Cattle	88	78	*A. bovis* (n = 1; 1.14%), *A. centrale* (n = 11; 12.50%), *A. marginale* (n = 25; 28.41%), *Anaplasma* spp. (n = 22; 25.00%), *Anaplasma* spp (n = 22; 25.00%), *A. platys* (n = 16; 18.18%), *B. bigemina* (n = 35; 39.77%), *B. rossi* (n = 1; 1.14%), *E. ruminatium* (n = 3; 3.41%), *T. velifera* (n = 69; 78.41%), *T. mutans* (n = 65; 73.86), *Theileria* spp. (n = 63; 71.59%)	[32]
Angola	Goats	82	2	*A. centrale* (n = 2; 2.44%)	[32]
Angola	Sheep	85	68	*A. centrale* (n = 2; 2.35%), *A. marginale* (n = 1; 1.18%), *Anaplasma* spp. (n = 4; 4.71%), *A. platys* (n = 5; 5.88%), *B. bovis* (n = 1; 1.18%), *T. ovis* (n = 68; 80.00%), *Theileria* spp. (n = 46; 54.12%)	[32]
Angola	Cattle	76	38	*B. bigemina* (n = 38; 50.00%)	[33]
Botswana	Cattle	276	2	*T. mutans* (n = 1; 0.36%), *T. taurotragi* (n = 1; 0.36%)	[34]
Botswana	Goats	100	76	*A. ovis* (n = 76; 76.00%)	[35]
Botswana	Cattle	429	135	*Anaplasma* spp. (n = 135; 31.47%)	[36]
Malawi	Goats	99	74	*A. ovis* (n = 61; 61.62%), *Anaplasma* spp. (n = 74; 74.75%)	[37]
Malawi	Sheep	8	8	*A. ovis* (n = 8; 100%), *Anaplasma* spp. (n = 8; 100%)	[37]
Mozambique	Cattle	219	213	*A. marginale* (n = 213; 97.26%), *A. phagocytophilum* (n = 6; 2.74%), *Anaplasma* spp. (n = 191; 87.21%)	[38]
Mozambique	Cattle	477	323	*A. centrale* (n = 20; 4.19%), *A. bovis* (n = 4; 0.84%), *A. marginale* (n = 42; 8.80%), *B. bigemina* (n = 267; 55.97%), *B. bovis* (n = 201; 42.14%), *Ehrlichia* spp. (n = 29; 6.08%), *T. mutans* (n = 250; 52.41%), *T. taurotragi* (n = 5; 1.05%), *T. velifera* (n = 255; 53.46%), *Theileria* spp. (n = 41; 8.59%)	[39]
Mozambique	Cattle	117	104	*B. bigemina* (n = 104; 88.89%), *B. bovis* (n = 97; 82.91%)	[40]
Mozambique	Cattle	210	31	*E. ruminatium* (n = 31; 14.76%)	[41]
Mozambique	Cattle	49	6	*B. bigemina* (n = 6; 12.24%)	[33]
South Africa	Cattle	66	51	*A. centrale* (n = 27; 40.91%), *A. marginale* (n = 51; 77.27%)	[42]
South Africa	Cattle	517	295	*A. centrale* (n = 88; 17.02%), *A. marginale* (n = 295; 57.06%)	[43]
South Africa	Cattle	200	54	*T. parva* (n = 54; 27.00%)	[44]
South Africa	Cattle	149	88	*A. marginale* (n = 88; 59.06%)	[45]
South Africa	Cattle	846	140	*T. parva* (n = 140; 16.55%)	[46]
South Africa	Cattle	109	57	*B. bigemina* (n = 24; 22.02%) *B. bovis* (n = 33; 30.27%)	[47]
South Africa	Goats	31	0	0.00	[47]
South Africa	Sheep	10	3	*T. ovis* (n = 3; 30.00%)	[47]
South Africa	Cattle	430	278	*B. bigemina* (n = 278; 64.65%), *B. bovis* (n = 151; 35.12%)	[48]
South Africa	Cattle	50	32	*B. bovis* (n = 32; 64.00%)	[49]
South Africa	Cattle	215 755	129	*A. marginale* (n = 129; 60.00%) *Anaplasma* spp. (n = 648; 85.83%)	[50]
South Africa	Cattle	74	39	*A. marginale* (n = 39; 52.70%), *B. bigemina* (n = 2; 2.70%), *Ehrlichia* spp. (n = 14; 18.92%), *T. taurotragi* (n = 26; 35.14%)	[51]
South Africa	Cattle	268	210	*B. bigemina* (n = 204; 76.12%), *B. bovis* (n = 95; 35.45%)	[52]
South Africa	Cattle	250	182	*A. marginale* (n = 182; 72.80%)	[53]
South Africa	Cattle	265	78	*T. parva* (n = 78; 29.43%)	[54]
South Africa	Cattle	70	55	*Anaplasma* spp. (n = 55; 78.57%)	[36]
South Africa	Goats	61	54	*A. ovis* (n = 28; 45.90%), *E. ruminatium* (n = 12; 19.67%), *T. ovis* (n = 14; 22.95%)	[55]
South Africa	Sheep	30	10	*A. ovis* (n = 5; 16.67%), *E. ruminatium* (n = 1; 3.33%), *T. ovis* (n = 4; 13.33%)	[56]
South Africa	Cattle	1723	48	*E. ruminatium* (n = 48; 2.79%)	[57]
South Africa	Goats	308	17	*E. ruminatium* (n = 17; 5.52%)	[57]
South Africa	Sheep	350	20	*E. ruminatium* (n = 20; 5.71%)	[57]
South Africa	Cattle	81	30	*B. bigemina* (n = 30; 37.04%)	[33]
South Africa	Cattle	170	106	*B. bigemina* (n = 6; 3.53%), *B. bovis* (n = 9; 5.29%), *T. parva* (n = 8; 4.71%), *T. taurotragi* (n = 89; 52.35%)	[57]
South Africa	Cattle	60	50	*B. rossi* (n = 1; 1.67%)*T. mutans* (n = 49; 81.67%), *T. parva* (n = 4; 6.67%), *T. taurotragi* (n = 1; 1.67%), *T. velifera* (n = 42; 70.00%)	[17]
Tanzania	Cattle	354	98	*T. parva* (n = 98; 27.68%)	[58]
Tanzania	Cattle	381	374	*T. parva* (n = 374; 98.16%)	[59]
Tanzania	Cattle	130	124	*T. parva* (n = 124; 95.38%)	[60]
Tanzania	Cattle	960	303	*T. parva* (n = 303; 31.56%)	[61]
Tanzania	Cattle	336	116	*T. parva* (n = 116; 34.52%)	[62]
Tanzania	Cattle	160	39	*T. parva* (n = 39; 24.38%)	[63]
Tanzania	Cattle	245	153	*A. marginale* (n = 39; 15.92%), *B. bigemina* (n = 43; 17.55%), *B. bovis* (n = 11; 4.49%), *T. mutans* (n = 105; 42.86%), *T. ovis* (n = 3; 1.22%), *T. parva* (n = 63; 25.71%), *T. taurotragi* (n = 70; 30.20%)	[64]
Tanzania	Cattle	236	152	*A. marginale* (n = 24; 10.17%), *B. bigemina* (n = 12; 5.08%), *B. bovis* (n = 5; 2.12%), *T. mutans* (n = 90; 38.14%), *T. parva* (n = 81; 34.32%), *T. taurotragi* (n = 73; 30.93%), *T. velifera* (n = 8; 3.39%)	[65]
Tanzania	Cattle	150	105	*T. parva* (n = 105; 70.00%)	[66]
Tanzania	Cattle	64	9	*Theileria* spp. (n = 9; 14.06%)	[67]
Zambia	Cattle	130	21	*B. bigemina* (n = 19; 21.11%) *B. bovis* (n = 2; 2.22%)	[68]
Zambia	Cattle	142	78	*T. parva* (n = 78; 54.93%)	[69]
Zambia	Cattle	472	79	*B. bigemina* (n = 76; 16.10%), *T. parva* (n = 3; 0.64%)	[70]
Zambia	Goats	53	0	0	[70]
Zambia	Cattle	579	181	*Anaplasma* spp. (n = 69; 11.92%), *E. ruminatium* (n = 5; 0.86%), *T. mutans* (n = 94; 16.23%), *T. parva* (n = 4; 0.69%), *T. taurotragi* (n = 4; 0.69%)	[71]
Zambia	Cattle	232	99	*B. bigemina* (n = 24; 10.34%), *T. mutans* (n = 11; 4.74%), *T. parva* (n = 23; 9.91%), *T. taurotragi* (n = 41; 17.67%)	[72]
Zambia	Cattle	299	259	*A. marginale* (n = 77; 25.75%), *B. bigemina* (n = 10; 3.34%), *B. bovis* (n = 23; 7.69%), *T. mutans* (n = 163; 54.52%), *T. parva* (n = 1; 0.33%), *T. velifera* (n = 153; 51.17%)	[73]
Zambia	Cattle	71	34	*A. marginale* (n = 34; 47.89%), *B. bigemina* (n = 16; 22.54%), *T. parva* (n = 16; 22.54%)	[74]
Zimbabwe	Cattle	94	33	*B. bigemina* (n = 33; 35.11%), *B. bovis* (n = 27; 28.72%)	[75]

**Table 2:** Sub-group analysis for infection rates of tick-borne pathogens associated with animal hosts, pathogen genera, diagnostic technique, study years and countries.

Risk Factors	Number of Studies	Pooled Prevalence Estimates			Measure of Heterogeneity		*Q-p*	Publication Bias
		Sample Size	No. of Positive	Prevalence 95% CI	*Q*	I^2^		Begg and Mazumdar Rank *p*-Value
Overall Animals	48	12693	5172	52.2 (43.9–60.3)	2820.792	98.33	0.609	0.065
Animal hosts								
Cattle	45	12693	5172	51.2 (42.9–59.4)	2491.04	98.23	0.779	0.056
Goats	8	663	236	29.9 (7.3–69.9)	252.68	97.23	0.325	0.310
Sheep	5	483	109	45.4 (9.4–87.0)	146.22	97.26	0.861	0.312
Genus *Anaplasma*								
*A. bovis*	2	565	5	0.88	-	-	-	-
*A. centrale*	4	1148	146	14.7 (5.9–32.0)	69.01	95.65	0.001	0.500
*A. marginale*	14	2982	1264	45.9 (31.3–61.3)	618.20	97.90	0.605	0.351
*A. phagocytophilum*	2	317	8	2.52	-	-	-	-
*Anaplasma* spp.	7	2216	1126	45.6 (17.9–76.3)	760.30	99.21	0.797	0.440
Genus *Babesia*								
*B. bigemina*	22	4393	1280	20.8 (12.4–32.6)	1007.80	97.92	0.000	0.068
*B. bovis*	14	2733	723	20.3 (12.7–30.9)	373.29	96.52	0.000	0.070
Genus *Ehrlichia*								
*E. ruminantium*	5	2936	118	4.2 (1.6–10.2)	74.03	94.60	0.000	0.500
*Ehrlichia* spp.	2	551	43	7.80	-	-	-	-
Genus *Theileria*								
*Theileria* spp.	1	64	9	14.06	-	-	-	-
*T. mutans*	10	2591	831	29.1 (17.5–44.4)	369.35	97.56	0.009	0.210
*T. parva*	20	6288	1712	25.0 (17.6–34.1)	687.51	97.24	0.000	0.097
*T. velifera*	6	1236	549	43.0 (26.4–61.4)	135.20	96.30	0.459	0.286
Diagnostic technique								
nPCR	14	3815	2006	61.5 (45.6–75.2)	799.92	98.38	0.155	0.104
PCR	28	5432	2291	43.6 (34.8–52.8)	863.936	96.88	0.172	0.376
qPCR	4	2534	475	31.0 (6.7–73.8)	537.17	99.44	0.393	0.248
RLB	7	1428	863	63.0 (42.0–80.0)	201.38	97.02	0.222	0.440
RT-PCR	2	1046	194	18.55	-	-	-	-
htPCR	1	117	86	73.50	-	-	-	-
Study year								
1990–2000	1	276	2	0.72	-	-	-	-
2001–2010	9	2023	1170	63.6 (49.1–75.9)	273.97	97.08	0.066	0.267
2011–2020	21	5085	2586	57.3 (46.4–67.6)	844.80	97.63	0.187	0.040
Study countries								
Angola	4	338	178	54.3 (21.9–83.5)	85.86	96.51	0.814	0.248
Botswana	2	705	137	19.43	-	-	-	-
Mozambique	5	1072	677	62.9 (25.3–89.5)	255.31	98.43	0.521	0.312
South Africa	18	5543	1922	52.2 (37.6–66.4)	1212.40	98.60	0.772	0.367
Tanzania	10	3016	1474	57.8 (42.2–72.0)	432.85	97.92	0.326	0.020
Zambia	7	1925	751	41.7 (24.1–61.7)	330.51	98.18	0.417	0.226
Zimbabwe	1	94	33	35.11	-	-	-	-

htPCR: High throughput qPCR; PCR: conventional polymerase chain reaction; nPCR: nested PCR; qPCR: real time polymerase chain reaction; RT-PCR: reverse transcription-polymerase chain reaction; RLB: reverse line blot hybridization.

**Table 3:** Characteristics of all eligible studies reporting the occurrence of tick-borne pathogens in ticks collected from domestic ruminants across the Southern African Developing Community region.

Countries	Hosts	Tick Species	Molecular Technique	Sample Size	Counts of Detected Pathogens in Ticks	Pathogens Detected (No. of Positives, Prevalence (%)	Reference
Angola	Cattle	*A. variegatum*, *R. decoloratus*	PCR	116	6	*R. africae* (n = 5; 4.31%), *T. mutans* (n = 1; 0.86%)	[30]
Angola	Cattle, goats, sheep	*R. compositus*	PCR, RLB	2963	43	*E. ruminatium* (n = 43; 1.45%)	[32]
Comoros	Cattle, Goats	*A. variegatum*, *R. appendiculatus*, *R.(B). microplus*	PCR	512	94	*R. africae* (n = 94; 18.36%)	[76]
Madagascar	Cattle, Goats	*H. simplex*, *R. microplus*	PCR	235	60	*R. africae* (n = 60; 26.67%)	[77]
Madagascar	Cattle	*A. variegatum*, *R. microplus*	PCR	499	312	*A. marginale* (n = 311; 62.32%), *A. ovis* (n = 1; 0.15%)	[78]
Mozambique	Cattle	*A. variegatum*, *R. microplus*	PCR	646	5	*R. africae* (n = 4; 0.62%) *T. velifera* (n = 1; 0.15%)	[79]
South Africa	Cattle, goats, sheep	*R. appendiculatus*, *R. decoloratus*, *R. e. evertsi*	PCR	1200	26	*E. ruminatium* (n = 19; 1.58%), *A. bovis* (n = 1; 0.25%), *A. marginale* (n = 2; 0.15%), *A. ovis* (n = 3; 0.33%), *B. caballi* (n = 1; 0.25%)	[80]
South Africa	Cattle, sheep	*A. hebraeum*, *H.m. rufipes*, *R. appendiculatus*, *R. (B.) decoloratus*, *R. e. evertsi*	PCR	7364	58	*B. bigemina* (n = 4; 0.31%), *Babesia* spp. (n = 1; 0.38%), *E. ruminatium* (n = 5; 2.15%), *E. ovina* (n = 2; 0.17%), *Ehrlichia* spp. (n = 8; 0.61%), *T. bicornis* (n = 7; 0.75%), *T. buffeli* (n = 7; 0.45%), *T. mutans* (n = 2; 0.18%), *T. ovis* (n = 2; 0.22%), *T. separata* (n = 4; 0.44%), *T. taurotragi* (n = 3; 0.32%), *Theileria* spp. (n = 13; 0.71%)	[81]
South Africa	Cattle, sheep	*A. hebraeum*, *R. appendiculatus*, *R. decoloratus*, *R. e. evertsi*	PCR	130	24	*A. marginale* (n = 5; 3.85%), *E. ruminatium* (n = 2; 1.54%), *Rickettsia* spp. (n = 10; 7.69%), *T. mutans* (n = 4; 3.08%), *T. taurotragi* (n = 3; 2.31%)	[82]
South Africa	Cattle, goats, sheep	*A. hebraeum*, *R. appendiculatus*, *R. decoloratus*, *R. e. evertsi*, *R. sanguineus*	PCR	760	16	*Ehrlichia* spp. (n = 16; 2.10%)	[83]
South Africa	Cattle, goats, sheep	*A. hebraeum*, *H. truncatum*, *R. appendiculatus*, *R. e. evertsi*, *R. microplus*, *R. simus*	PCR	903	60	*Rickettsia* spp. (n = 60; 6.64%)	[84]
South Africa	Cattle	*R. sanguineus*	PCR	100	10	*A. phagocytophilum* (n = 10; 10%)	[85]
South Africa	Cattle, goats, sheep	*A. hebraeum*	PCR	1403	344	*E. ruminatium* (n = 344; 24.52%)	[57]
South Africa	Goats	*A. hebraeum*	PCR	630	47	*E. ruminatium* (n = 19; 3.02%) *R. africae* (n = 28; 4.44%)	[86]
Tanzania	Cattle, Goats	-	PCR	819	0	-	[87]
Tanzania	Cattle	*A. gemma*, *R. appendiculatus*, *R. praetextatus*, *R. pulchellus*	PCR	527	28	*A. marginale* (n = 28; 5.31%)	[88]
Tanzania	Cattle	*A. gemma*, *A. lepidum*, *A. marmoreum*, *A. variegatum*, *H. impeltatum*, *R. pulchellus*	PCR	263	160	*Babesia* spp. (n = 7; 2.66%), *Ehrlichia* spp. (n = 6; 2.28%), *Rickettsia* spp. (n = 133; 50.57%), *Theileria* spp. (n = 14; 5.32%)	[89]
Zambia	Cattle	*A. variegatum*	RLB	5288	1	*E. ruminatium* (n = 1; 0.02%)	[73]
Zambia	Cattle	*R. appendiculatus*	PCR	74	10	*T. parva* (n = 10; 13.51%)	[74]
Zimbabwe	Cattle	*H. truncatum*, *R. e. evertsi*	PCR	1141	288	*E. ruminatium* (n = 288; 25.24%)	[90]
Zimbabwe	Cattle	*R. appendiculatus*	PCR	36	18	*B. bigemina* (n = 12; 33.33%), *B. bovis* (n = 6; 16.67%)	[75]

**Table 4:** Sub-group analysis for infection rates of tick-borne pathogens detected in ticks collected from different domestic ruminants.

Risk Factors	Number of Studies	Pooled Prevalence Estimates			Measure of Heterogeneity		*Q-p*	Publication Bias
		Sample Size	Number of Positive	Prevalence 95% CI (%)	*Q*	I^2^		Begg and Mazumdar Rank *p*-Value
Overall ticks	20	18355	1601	7.7 (4.0–14.3)	2310.69	99.18	0.000	0.060
Genus *Anaplasma*								
*A. marginale*	4	2428	348	6.8 (0.6–45.2)	333.05	99.10	0.034	0.248
Genus *Ehrlichia*								
*E. ruminantium*	8	3719	701	4.6 (2.2–9.1)	347.46	97.98	0.000	0.161
*Ehrlichia* spp.	3	1543	31	2.1 (1.4–3.3)	3.02	33.72	0.000	0.301
Genus *Rickettsia*								
*R. africae*	5	978	185	18.0 (7.4–37.5)	104.23	96.16	0.003	0.164
*Rickettsia* spp.	3	859	203	39.0 (4.0–90.8)	136.03	98.53	0.749	0.059
Genus *Theileria*								
*T. mutans*	3	1193	7	2.6 (0.2–31.2)	23.58	91.52	0.012	0.301

**Table 5:** Pooled prevalence estimates and risk factor associated with ticks species and tick-borne pathogen infections in animal ticks.

Risk Factors	Number of Studies	Pooled Prevalence Estimates			Measure of Heterogeneity		*Q-p*	Publication Bias
		Sample Size	No. of Positive	Prevalence95% CI (%)	*Q*	I^2^		Begg and Mazumdar Rank *p*-Value
Genus *Amblyomma*	15	3987	959	25.0 (14.7–39.1)	598.25	97.66	0.001	0.200
*A. chabaudi*	1	2	2	100.00	-	-	-	-
*A. gemma*	2	79	20	25.32				
*A. hebraeum*	7	2344	456	14.2 (8.9–21.8)	64.23	90.66	0.000	0.440
*A. lepidum*	1	42	8	19.05	-	-	-	-
*A. marmoreum*	1	11	2	18.18	-	-	-	-
*A. pomposum*	1	617	43	6.97	-	-	-	-
*A. variegatum*	7	3713	431	43.9 (10.1–84.4)	250.42	97.60	0.804	0.440
Genus *Haemaphysalis*	1	19	2	10.53	-	-	-	-
*H. simplex*	1	19	2	10.53	-	-	-	-
Genus *Hyalomma*	2	909	119	13.1	-	-	-	-
*H.m. rufipes*	2	582	89	15.29	-	-	-	-
*H. truncatum*	1	327	20	6.12	-	-	-	-
Genus *Rhipicephalus*	14	8730	522	8.0 (3.2–18.6)	841.80	98.46	0.000	0.162
*R. appendiculatus*	9	899	40	3.7 (1.6–8.3)	46.25	82.70	0.000	0.266
*R. (B.) decoloratus*	3	424	36	36.9 (3.1–91.5)	63.91	96.87	0.000	0.30
*R. compositus*	2	181	7	3.87	-	-	-	-
*R. decoloratus*	2	42	12	28.57	-	-	-	-
*R.(B). microplus*	2	312	14	4.49	-	-	-	-
*R. e. evertsi*	5	1718	234	7.4 (1.1–35.8)	317.76	98.74	0.011	0.312
*R. microplus*	3	693	174	15.4 (1.1–75.5)	173.07	98.84	0.238	0.301
*R. praetextatus*	1	23	2	8.70	-	-	-	-
*R. pulchellus*	1	22	6	27.27	-	-	-	-
*R. sanguineus*	2	280	15	5.36	-	-	-	-

**Table 6:** Search strategies.

S/No.	Source	Query/Search String	Results
1	PubMed	Ticks and tick-borne pathogens in Southern Africa; Prevalence of “Anaplasma” “Babesia” “Ehrlichia” and/or “Theileria”	56
2	Science direct	Ticks and tick-borne pathogens in Southern Africa; Prevalence of “Anaplasma” “Babesia” “Ehrlichia” and/or “Theileria”	751
3	Google scholar	Ticks and tick-borne pathogens in Southern Africa; Prevalence of “Anaplasma” “Babesia” “Ehrlichia” and/or “Theileria”	31,700
4	AJOL	Ticks and tick-borne pathogens in Southern Africa; Prevalence of “Anaplasma” “Babesia” “Ehrlichia” and/or “Theileria”	244
5	Springer Link	Ticks and tick-borne pathogens in Southern Africa; Prevalence of “Anaplasma” “Babesia” “Ehrlichia” and/or “Theileria”	743

S/No. = Searching number.

## Data Availability

The data presented in this study are available on request from the corresponding author.

## Supplementary Materials

The following supporting information can be downloaded at: https://www.mdpi.com/article/10.3390/pathogens11080929/s1, Figure S1: Forest plot showing the pooled estimates of *Anaplasma marginale* in South Africa. The squares demonstrate the individual point estimate. The diamond at the base indicates the pooled estimates from the overall studies. Figure S2: Forest plot showing the prevalence of *Babesia bigemina* in South Africa. The squares demonstrate the individual point estimate. The diamond at the base indicates the pooled estimates from the overall studies. Figure S3: Forest plot showing the pooled estimates of *Babesia bovis* in South Africa. The squares demonstrate the individual point estimate. The diamond at the base indicates the pooled estimates from the overall studies. Forest plot showing the prevalence of *Theileria parva* in South Africa. Figure S4: The squares demonstrate the individual point estimate. The diamond at the base indicates the pooled estimates from the overall studies.
