# Supplementary material for: Epidemiology of Ticks and Tick-Borne Pathogens in Domestic Ruminants across Southern African Development Community (SADC) Region from 1980 until 2021: A Systematic Review and Meta-Analysis
Source: Pathogens. 2022 Aug 18;11(8):929. doi: 10.3390/pathogens11080929 (PMC9414594; doi:10.3390/pathogens11080929)
Supplement: Supplementary file 1 [file pathogens-11-00929-s001.zip › pathogens-1645642-supplementary.pdf]

Supplementary Materials

Meta Analysis

Study name	Subgroup within study	Event rate	Lower limit	Upper limit	Z-Value	p-Value
Chaisi et al., 2017	*A. marginale*	0.773	0.657	0.858	4.166	0.000
de Jesus Fernandes et al., 2019	*A. marginale*	0.973	0.940	0.988	8.623	0.000
Hove et al., 2018	*A. marginale*	0.571	0.528	0.613	3.200	0.001
Kubelová et al., 2012	*A. marginale*	0.382	0.280	0.495	-2.045	0.041
Martins et al., 2010	*A. marginale*	0.088	0.066	0.117	-14.468	0.000
Marufu et al., 2012	*A. marginale*	0.591	0.510	0.667	2.200	0.028
Mtshali et al., 2007	*A. marginale*	0.600	0.533	0.663	2.913	0.004
Mtshali et al., 2013	*A. marginale*	0.486	0.375	0.599	-0.232	0.816
Mutshembele et al., 2014	*A. marginale*	0.728	0.670	0.780	6.927	0.000
Ringo et al., 2018	*A. marginale*	0.159	0.119	0.210	-9.531	0.000
Ringo et al., 2020	*A. marginale*	0.102	0.069	0.147	-10.115	0.000
Sili et al., 2021	*A. marginale*	0.284	0.200	0.387	-3.910	0.000
Tembo et al., 2018	*A. marginale*	0.258	0.211	0.310	-8.006	0.000
Yamada et al., 2009	*A. marginale*	0.479	0.366	0.594	-0.356	0.722
		0.438	0.417	0.460	-5.592	0.000

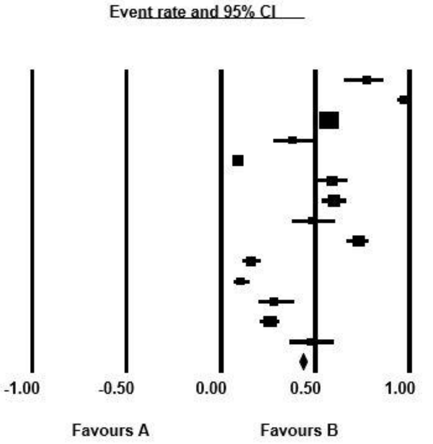

Meta Analysis

Figure S1: Forest plot showing the pooled estimates of *Anaplasma marginale* in South Africa. The squares demonstrate the individual point estimate. The diamond at the base indicates the pooled estimates from the overall studies

Meta Analysis

Study name	Subgroup within study	Event rate	Lower limit	Upper limit	Z-Value	p-Value
Mtshali and Mtshali, 2013	*B. bigemina*	0.647	0.600	0.690	5.985	0.000
Mtshali et al., 2013	*B. bigemina*	0.027	0.007	0.102	-4.999	0.000
Mtshali et al., 2013b	*B. bigemina*	0.761	0.706	0.808	8.091	0.000
Thompson et al., 2008	*B. bigemina*	0.035	0.016	0.076	-7.959	0.000
Yusufmia et al., 2010	*B. bigemina*	0.017	0.002	0.109	-4.043	0.000
Mofokeng et al., 2020	*B. bigemina*	0.220	0.152	0.308	-5.471	0.000
Stoltsz et al., 2020a	*B. bigemina*	0.370	0.273	0.480	-2.306	0.021
Kubelová et al., 2012	*B. bigemina*	0.026	0.007	0.099	-5.039	0.000
Sili et al., 2021	*B. bigemina*	0.909	0.829	0.954	6.210	0.000
Martins et al., 2010	*B. bigemina*	0.560	0.515	0.604	2.604	0.009
Martins et al., 2008	*B. bigemina*	0.889	0.818	0.934	7.069	0.000
Ringo et al., 2018	*B. bigemina*	0.176	0.133	0.228	-9.212	0.000
Ringo et al., 2020	*B. bigemina*	0.051	0.029	0.087	-9.877	0.000
Squarre et al., 2020	*B. bigemina*	0.103	0.070	0.150	-10.017	0.000
Musinguzi et al., 2016	*B. bigemina*	0.161	0.131	0.197	-13.181	0.000
Simuunza et al., 2011	*B. bigemina*	0.009	0.004	0.021	-10.560	0.000
Tembo et al., 2018	*B. bigemina*	0.033	0.018	0.061	-10.458	0.000
Smeenk et al., 2000	*B. bigemina*	0.351	0.262	0.452	-2.843	0.004
Yamada et al., 2009	*B. bigemina*	0.225	0.143	0.337	-4.347	0.000
Iseki et al., 2007	*B. bigemina*	0.211	0.139	0.307	-5.104	0.000
Stoltsz et al., 2020b	*B. bigemina*	0.122	0.056	0.247	-4.519	0.000
Stoltsz et al., 2020c	*B. bigemina*	0.500	0.389	0.611	0.000	1.000
		0.393	0.373	0.413	-10.244	0.000

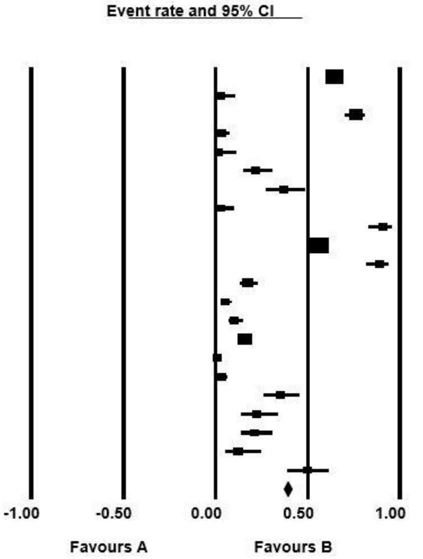

Meta Analysis

Figure S2: Forest plot showing the prevalence of *Babesia bigemina* in South Africa. The squares demonstrate the individual point estimate. The diamond at the base indicates the pooled estimates from the overall studies

Meta Analysis

Study name	Subgroup within study	Event rate	Lower limit	Upper limit	Z-Value	p-Value
Mtshali and Mtshali, 2013	*B. bovis*	0.351	0.307	0.397	-6.077	0.000
Mtshali and Mtshali, 2017	*B. bovis*	0.640	0.499	0.760	1.953	0.051
Mtshali et al., 2013	*B. bovis*	0.354	0.299	0.414	-4.694	0.000
Thompson et al., 2008	*B. bovis*	0.053	0.028	0.099	-8.420	0.000
Yusufmia et al., 2010	*B. bovis*	0.033	0.008	0.124	-4.682	0.000
Mofokeng et al., 2020	*B. bovis*	0.303	0.224	0.395	-4.002	0.000
Sili et al., 2021	*B. bovis*	0.398	0.301	0.503	-1.905	0.057
Martins et al., 2010	*B. bovis*	0.421	0.378	0.466	-3.420	0.001
Martins et al., 2008	*B. bovis*	0.829	0.750	0.887	6.430	0.000
Ringo et al., 2018	*B. bovis*	0.045	0.025	0.079	-9.910	0.000
Ringo et al., 2020	*B. bovis*	0.021	0.009	0.050	-8.480	0.000
Tembo et al., 2018	*B. bovis*	0.077	0.052	0.113	-11.450	0.000
Smeenk et al., 2000	*B. bovis*	0.287	0.205	0.386	-3.987	0.000
Iseki et al., 2007	*B. bovis*	0.022	0.006	0.085	-5.292	0.000
		0.333	0.312	0.355	-14.146	0.000

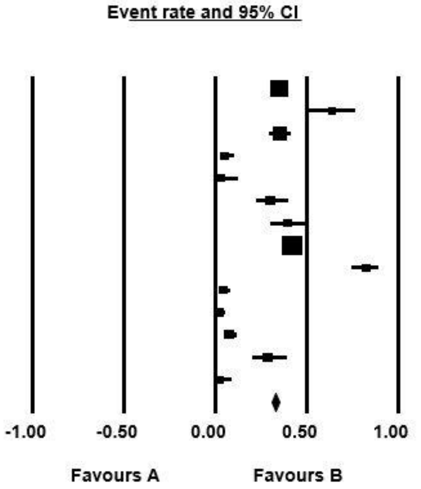

Meta Analysis

Figure S3: Forest plot showing the pooled estimates of *Babesia bovis* in South Africa. The squares demonstrate the individual point estimate. The diamond at the base indicates the pooled estimates from the overall studies

Meta Analysis

Study name	Subgroup within study	Event rate	Lower limit	Upper limit	Z-Value	p-Value
Muleya et al., 2012	*T. parva*	0.549	0.467	0.629	1.173	0.241
Kazungu et al., 2015	*T. parva*	0.982	0.962	0.991	10.429	0.000
Kerario et al., 2019	*T. parva*	0.954	0.901	0.979	7.245	0.000
Kimaro et al., 2017	*T. parva*	0.316	0.287	0.346	-11.145	0.000
Laisser et al., 2014	*T. parva*	0.277	0.233	0.326	-8.083	0.000
Latif et al., 2019	*T. parva*	0.270	0.213	0.336	-6.245	0.000
Magulu et al., 2019	*T. parva*	0.345	0.296	0.398	-5.578	0.000
Mbizeni et al., 2013	*T. parva*	0.165	0.142	0.192	-17.488	0.000
Musinguzi et al., 2016	*T. parva*	0.006	0.002	0.020	-8.722	0.000
Mwamuye et al., 2020	*T. parva*	0.244	0.183	0.316	-6.149	0.000
Pienaar et al., 2020	*T. parva*	0.294	0.243	0.352	-6.487	0.000
Ringo et al., 2018	*T. parva*	0.257	0.206	0.316	-7.257	0.000
Ringo et al., 2020	*T. parva*	0.343	0.285	0.406	-4.733	0.000
Rukambile et al., 2016	*T. parva*	0.700	0.622	0.768	4.755	0.000
Simuunza et al., 2011	*T. parva*	0.007	0.003	0.018	-9.902	0.000
Squarre et al., 2020	*T. parva*	0.099	0.067	0.145	-10.045	0.000
Tembo et al., 2018	*T. parva*	0.003	0.000	0.023	-5.688	0.000
Thompson et al., 2008	*T. parva*	0.047	0.024	0.091	-8.306	0.000
Yamada et al., 2009	*T. parva*	0.225	0.143	0.337	-4.347	0.000
Yusufmia et al., 2010	*T. parva*	0.067	0.025	0.165	-5.099	0.000
		0.293	0.279	0.308	-25.288	0.000

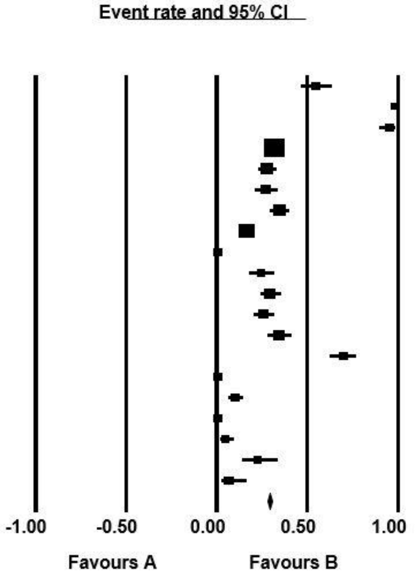

Meta Analysis

Figure S4: Forest plot showing the prevalence of *Theileria Parva* in South Africa. The squares demonstrate the individual point estimate. The diamond at the base indicates the pooled estimates from the overall studies
